# Inadvertent Salbutamol Overdose Presenting as Acute Toxicity

**DOI:** 10.7759/cureus.95282

**Published:** 2025-10-24

**Authors:** Shaikh Mohammed Aslam, Aadithya Shyllesh H, Vaibhav Siddarth TS, Mohammed Suhail K, Ashwin Kulkarni

**Affiliations:** 1 Internal Medicine, Ramaiah Medical College and Hospital, Ramaiah University of Applied Sciences, Bengaluru, IND

**Keywords:** asthma, drug-induced hypokalemia, salbutamol-induced lactic acidosis, salbutamol overdose, salbutamol toxicity

## Abstract

Salbutamol is a short-acting beta-2 agonist available in inhalational and oral formulations for the treatment of asthma and chronic obstructive pulmonary disease due to its ability to rapidly relieve bronchospasm. However, because of its quick onset of action, it is recommended only as rescue therapy in mild cases with minimal symptoms and without nocturnal awakenings. Owing to its efficacy, salbutamol carries a potential risk of misuse or overuse among asthmatic patients. We report the case of a young woman with a long-standing history of asthma who presented with seizures followed by loss of consciousness after consuming multiple salbutamol tablets in succession. Neuroimaging (MRI brain) and electroencephalogram findings were normal. Further evaluation revealed cardiac arrhythmias, metabolic dysfunction characterized by hyperglycemia and lactic acidosis, and hypokalemia. She was managed conservatively in the ICU. After stabilization, her asthma medications were optimized, and she was discharged with education on the appropriate use of salbutamol to prevent recurrence of toxicity.

## Introduction

Salbutamol is a short-acting beta-2 adrenergic receptor agonist. Inhalational salbutamol is commonly recommended for the treatment of asthma and other chronic obstructive pulmonary diseases, as it induces smooth muscle relaxation in the airways, thereby reversing bronchoconstriction. Side effects such as tremors, hyperglycemia (or hyperinsulinemia), hypokalemia, hyperlactatemia, hypotension, tachycardia with palpitations, QTc prolongation, ventilation mismatch, and increased metabolic rate may occur due to stimulation of both beta-1 and beta-2 receptors by salbutamol [1].

The pharmacokinetics of salbutamol depend on its formulation (oral or IV) and delivery mechanism (metered-dose inhaler (MDI) or dry powder inhaler), which influence drug absorption, airway deposition, effectiveness, and side effect profile. Following inhalation, salbutamol primarily acts on bronchial smooth muscles with minimal systemic effects. After two to three hours, low plasma concentrations are observed due to the swallowing and oral absorption of the inhaled drug. Oral administration is rapidly and efficiently absorbed, reaching peak plasma concentrations approximately two hours after ingestion. However, the drug undergoes a significant first-pass effect because of extensive hepatic metabolism and intestinal mucosal absorption, resulting in about 50% bioavailability. Owing to its large volume of distribution, salbutamol is not considered dialyzable.

Inhaled salbutamol, administered via a spacer or inhaler, is preferred due to its rapid onset of action, reduced severity of adverse effects, and ease of administration, making it a cornerstone in the management of severe acute asthma. It is typically administered every 20 minutes during initial treatment at a dose of 0.05-0.15 mg/kg, with a maximum of 20 mg per hour. Inhalational salbutamol is also used in obstetrics as a tocolytic agent and as an adjunct treatment for severe hyperkalemia. IV salbutamol serves as a second- or third-line treatment option in severe acute asthma. However, oral salbutamol is not recommended during asthma exacerbations because it has a slower onset of action, greater incidence of side effects, and no additional benefit compared with inhaled formulations. It is sometimes used in combination with nebulized salbutamol.

Given its mechanism of action, it is important to recognize that short-acting beta-agonist (SABA) monotherapy with salbutamol is intended only as a reliever medication to rapidly reverse acute asthma exacerbations. SABA-only treatment can lead to poor asthma control, adverse clinical outcomes, and potential psychological dependence, overuse, and toxicity. Long-acting beta-2 agonists, antimuscarinic agents, and inhaled corticosteroids are essential controller medications in the proper management of asthma. According to the International Association of Forensic Toxicologists, the therapeutic plasma concentration of salbutamol ranges between 4 and 18 ng/mL, with toxic levels above 30 ng/mL and lethal levels exceeding 160 ng/mL. Neuropsychiatric effects, including antidepressant-like properties and hallucinations, have been reported with excessive use of the drug [2].

We report the case of a young woman with poorly controlled asthma who was receiving salbutamol-only treatment. Inadequate symptom control led to inadvertent overuse of the drug, and she presented with clinical features consistent with salbutamol toxicity.

## Case presentation

A young woman with a known history of hypothyroidism and bronchial asthma presented to the emergency department with a history of one episode of giddiness, a fall, and involuntary movements involving all four limbs, followed by loss of consciousness lasting less than five minutes. Upon arrival, she was conscious and oriented. On further inquiry, she admitted to having voluntarily and successively consumed 20 tablets of salbutamol in a desperate attempt to relieve worsening wheezing that had progressively intensified since earlier that morning.

On examination, the patient was tachycardic with a heart rate of 120 beats per minute, tachypneic with a respiratory rate of 33 cycles per minute, and hypoxic with an oxygen saturation of 91% on room air, requiring 1-2 liters of oxygen via nasal prongs. Her random blood glucose level on admission was 243 mg/dL. Chest auscultation revealed clear lung fields. The remainder of her systemic examination was unremarkable.

Blood investigations revealed lactic acidosis and hypokalemia. Her hypothyroidism was well controlled with thyroxine 25 mcg/day, and thyroid function parameters were within normal limits (Table 1). Renal and hepatic function tests, as well as coagulation profiles, were also within normal ranges.

**Table 1:** Laboratory parameters at presentation Alb, albumin; ALT, alanine aminotransferase; ALP, alkaline phosphatase; APTT, activated partial thromboplastin time; AST, aspartate aminotransferase; B, basophils; BUN, blood urea nitrogen; CXR, chest X-ray; DB, direct bilirubin; DLC, differential leukocyte count; EF, ejection fraction; EEG, electroencephalogram; E, eosinophils; ESR, erythrocyte sedimentation rate; Free T₃, free triiodothyronine; Free T₄, free thyroxine; GGT, gamma-glutamyl transferase; GRBS, gross random blood sugar; Hb, hemoglobin; HCO₃⁻, bicarbonate; IVC, inferior vena cava; INR, international normalized ratio; L, lymphocytes; LV, left ventricle; M, monocytes; N, neutrophils; PASP, pulmonary artery systolic pressure; PCV, packed cell volume; PLT, platelets; PT, prothrombin time; QT, QT interval; QTc, corrected QT interval; RWMA, regional wall motion abnormality; TB, total bilirubin; TLC, total leukocyte count; TP, total protein; Trop-T, troponin T; TSH, thyroid-stimulating hormone

Parameters	Values
Complete blood count	Hb - 10.5 g/dL; PCV - 37.4%; TLC - 19.85 × 10⁹/L; DLC: N - 89.3%, L - 5.6%, M - 4.8%, E - 0.1%, B - 0.2%; PLT - 367 × 10⁹/L
Renal parameters	Creatinine - 0.697 mg/dL; BUN - 7.22 mg/dL; uric acid - 5.46 mg/dL; sodium - 140.6 mEq/L; potassium - 2.85 mEq/L; chloride - 101.9 mEq/L; phosphorus - 2.03 mg/dL; calcium - 8.39 mg/dL
Coagulation profile	PT - 13.2 seconds; APTT - 27.5 seconds; INR - 1.09
Inflammatory markers	ESR - 24 mm/hr; CRP - 1.49 mg/dL
Cardiac markers	Trop-T - 7.78 pg/mL; CK-MB - 29.4 U/L
Arterial blood gas	pH - 7.318; pCO₂ - 42.83 mmHg; pO₂ - 64 mmHg; HCO₃⁻ - 20.3 mEq/L; SO₂ - 88%; Lactate - 6.3 mEq/L
Endocrine parameters	GRBS - 243 mg/dL; TSH - 4.23 µIU/mL; Free T₄ - 1.2 ng/dL; Free T₃ - 2.4 pg/mL
Liver parameters	TB/DB - 0.7/0.2 mg/dL; TP/Alb - 6.7/4.12 g/dL; AST/ALT - 18/11 U/L; ALP/GGT - 49.5/3.8 U/L
CXR	Normal
ECG	Sinus tachycardia with QT prolongation
2D ECHO	Normal LV systolic function; EF - 56%; no RWMA; PASP - 35 mmHg; IVC - normal size and collapsible
MRI brain	Normal study
EEG	Right posterior slowing noted

The ECG revealed sinus tachycardia with QT prolongation, likely secondary to hypokalemia. The corrected QT interval (QTc) was calculated using Bazett’s formula:

$QT_c = \frac{QT}{\sqrt{RR}},$

where QT is the measured QT interval and RR is the RR interval, both expressed in seconds.

**Figure 1:**
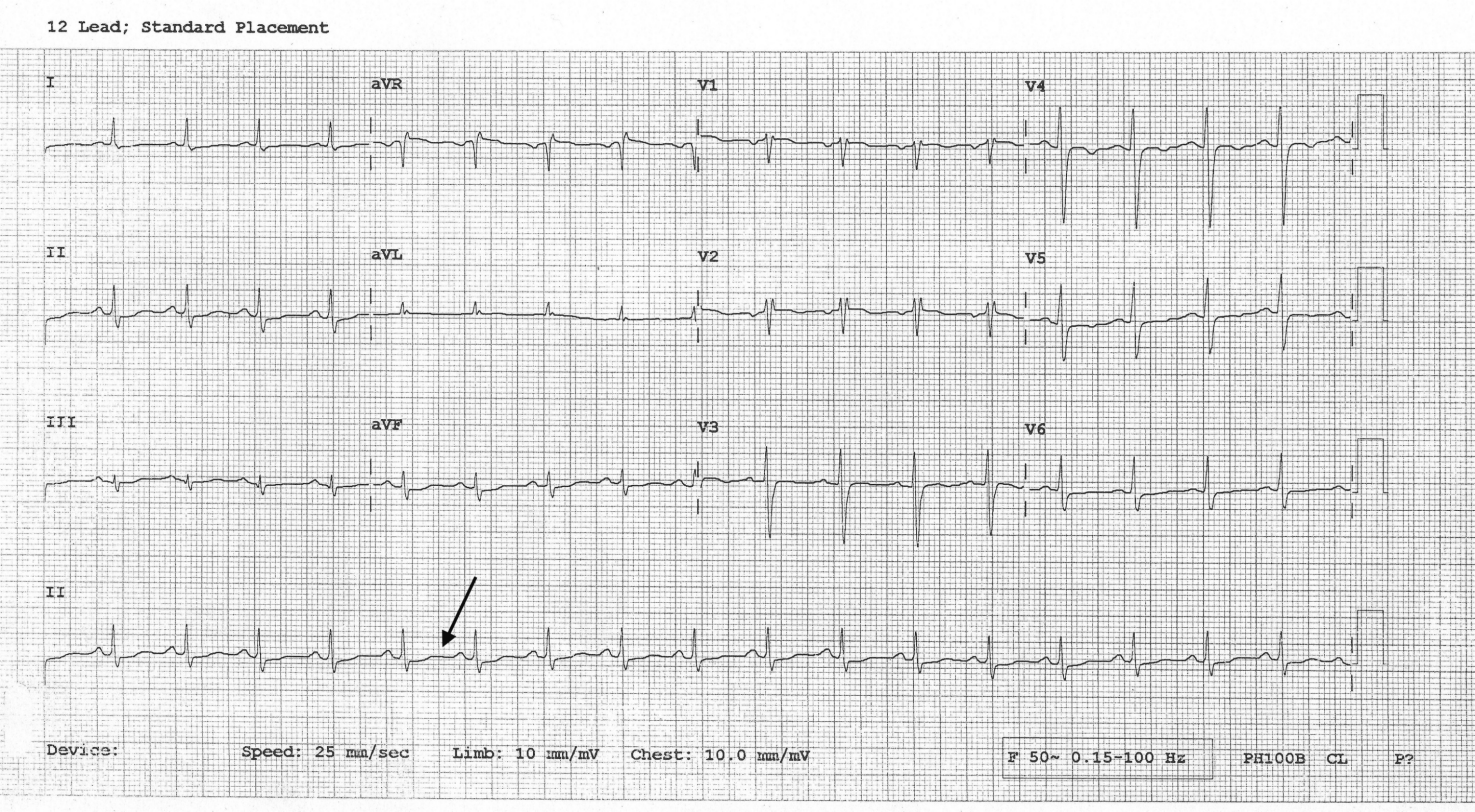
ECG showing sinus tachycardia with QT prolongation (QTc = 566 ms) QT, QT interval; QTc, corrected QT interval

Cardiac enzyme levels were elevated at presentation but normalized on subsequent testing. Echocardiography findings were not suggestive of an acute cardiac event (Figure 1).

The patient was decontaminated with activated charcoal and managed with IV fluids and potassium supplementation. Her serum electrolyte and blood lactate levels subsequently normalized. Neuroimaging with MRI brain and EEG findings were within normal limits. Further evaluation ruled out any suicidal intent or underlying psychiatric illness. Her asthma medications were optimized, and she was counseled on the appropriate dosage and frequency of salbutamol use to prevent future episodes of overuse or toxicity.

## Discussion

Salbutamol serves as a cornerstone in the management of acute bronchoconstriction in conditions such as asthma. Its rapid onset of action and potent bronchodilatory properties render it invaluable for reversing airflow obstruction in both emergency and domiciliary settings [3]. While generally considered safe within therapeutic ranges, exceeding recommended doses can precipitate a spectrum of adverse systemic effects, collectively termed salbutamol toxicity. The clinical presentation of this toxicity can be diverse, encompassing cardiovascular, metabolic, and neurological disturbances. The complexity of managing such an event intensifies when considering patients with comorbidities, particularly endocrine disorders that modulate adrenergic responsiveness.

For a young female patient concurrently presenting with asthma and hypothyroidism, the physiological interactions between these conditions and salbutamol's pharmacodynamics warrant meticulous consideration. Hypothyroidism, known to influence adrenergic sensitivity, may alter both the therapeutic efficacy and the toxicological profile of β2-agonists [4]. Understanding these intricate relationships is essential for accurate diagnosis and effective management of salbutamol overdose in this specific patient demographic. This article examines the pharmacodynamics of salbutamol, its therapeutic and toxicological thresholds, the systemic and neurological sequelae of overdose, and the specific influence of hypothyroidism on its toxicity, culminating in a discussion of diagnostic and therapeutic strategies.

Salbutamol functions as a synthetic sympathomimetic agent, exerting its primary therapeutic action through selective agonism of β2-adrenergic receptors. These receptors are predominantly located on the smooth muscle cells of the airways, where their activation triggers a cascade of intracellular events. Upon binding, salbutamol initiates the stimulation of adenyl cyclase, an enzyme responsible for catalyzing the conversion of adenosine triphosphate to cyclic adenosine monophosphate (cAMP) [5]. Elevated intracellular cAMP levels subsequently activate protein kinase A, which then phosphorylates various target proteins. This phosphorylation leads to a reduction in intracellular calcium concentrations, ultimately promoting relaxation of bronchial smooth muscle and consequently bronchodilation.

Beyond its beneficial effects on the respiratory system, β2-adrenergic receptors are distributed throughout the body, including the heart, skeletal muscle, liver, and pancreas. Consequently, salbutamol administration can elicit systemic effects that extend beyond the lungs. Activation of cardiac β2-receptors contributes to increased heart rate and myocardial contractility [6]. In skeletal muscle, β2-receptor stimulation promotes potassium uptake, which can lead to hypokalemia [7]. Furthermore, hepatic glycogenolysis and pancreatic insulin release can be stimulated, affecting glucose metabolism [7]. These widespread systemic actions underscore the potential for adverse effects, particularly with higher doses or prolonged exposure.

Therapeutic dosing of salbutamol is meticulously calibrated to maximize bronchodilation while minimizing systemic side effects. For acute exacerbations of asthma, typical inhaled doses via MDI range from 100 to 200 μg, administered as needed [8]. These doses often provide rapid relief from bronchospasm. When administered via nebulizer, common doses for adults and older children are 2.5-5 mg per treatment, which can be repeated every 20 minutes for severe exacerbations. The nebulized route delivers a larger total dose to the airways compared to MDI, making it suitable for more severe presentations. Studies have confirmed the effectiveness of nebulized salbutamol in improving lung function parameters such as forced expiratory volume in one second and peak expiratory flow [8].

However, the concept of a “standard dose” also accounts for individual patient variability and the severity of their respiratory distress. Pediatric dosing, for instance, is often weight-adjusted, and clinicians frequently titrate therapy based on clinical response. Despite its widespread use, even intermittent nebulized treatment at what are considered standard therapeutic doses can occasionally lead to adverse reactions, underscoring the necessity of vigilant monitoring during administration. The judicious application of these guidelines aims to achieve optimal therapeutic outcomes without eliciting an unacceptable burden of systemic effects.

Salbutamol toxicity occurs when exposure exceeds the body’s capacity to metabolize or tolerate the drug, leading to pronounced pharmacological effects in nontarget systems. While precise “lethal doses” are ill-defined in clinical practice due to the rarity of fatalities from isolated salbutamol overdose, toxic thresholds are well-documented. An instance involving a 12-year-old boy experiencing toxicity highlights this: symptoms manifested after an overall nebulized dose of 11.25 mg within an hour in the emergency department, following prior intermittent inhaled doses of 1.4 mg over 24 hours [4]. This observation underscores that intermittent, standard-dose nebulized therapy can also be hazardous due to its infrequency of severe reactions [4].

The route of administration significantly influences systemic exposure and thus the risk of toxicity. IV administration, while offering rapid and complete systemic bioavailability, carries a higher propensity for adverse effects compared to inhaled or nebulized routes. For example, IV salbutamol infusions at 10 μg/min in healthy subjects caused significant changes in plasma potassium and glucose [3]. Risk factors for toxicity include preexisting cardiac conditions, renal impairment, concomitant use of other sympathomimetics, and conditions that alter adrenergic receptor sensitivity. Children, particularly those receiving high-dose continuous or intermittent nebulized treatments, represent a vulnerable population requiring careful monitoring for early signs of toxicity [4]. The transition from therapeutic benefit to toxic manifestation is often dose-dependent, emphasizing the importance of individualized patient assessment and adherence to prescribed regimens.

Salbutamol toxicity generates a range of systemic adverse effects, primarily due to widespread β2-adrenergic receptor stimulation. Cardiovascular complications constitute a prominent concern. Tachycardia, a common finding, arises from direct cardiac β2-receptor activation [9]. While many studies indicate no significant effect on myocardial ischemia or arrhythmias at therapeutic doses, overdose can induce severe arrhythmias, including supraventricular tachycardia and ventricular ectopy [9]. ECG alterations, such as T-wave flattening and QTc interval prolongation, have been observed even with cumulative inhaled doses. Paradoxical sustained diastolic hypotension has been reported in cases of toxicity.

Metabolic disturbances are also frequent. Hypokalemia, resulting from β2-mediated intracellular shift of potassium, is a characteristic feature of salbutamol overdose. Plasma potassium levels can significantly decrease, as demonstrated in a case where a 12-year-old boy's potassium dropped to 2.6 mEq/L. Hyperglycemia, a consequence of increased glycogenolysis and gluconeogenesis, is another common metabolic derangement. Lactic acidosis, with lactate levels reaching 8.1 mmol/L in one reported case, indicates profound metabolic dysfunction. Furthermore, salbutamol can dose-dependently increase resting metabolic rate [10]. These systemic effects underscore the broad physiological impact of salbutamol toxicity, extending beyond its intended bronchodilatory action.

Overdosing on salbutamol can induce a range of neurological and neuropsychiatric manifestations, primarily reflecting the drug's sympathomimetic effects on the central nervous system. Tremor, particularly fine motor tremor, is a commonly reported and often one of the earliest signs of systemic β2-adrenergic stimulation, even at therapeutic doses. This effect results from the activation of β2-receptors in skeletal muscles. With increasing toxicity, the tremor can become more pronounced and distressing for the patient.

Beyond tremor, patients may experience a spectrum of other neurological complications. Restlessness, anxiety, and agitation are frequently observed, reflecting central nervous system excitation. These symptoms can exacerbate feelings of discomfort and distress, particularly in a patient already experiencing respiratory distress from an asthma exacerbation. While less common, severe overdose may precipitate more serious neurological events such as seizures. These complications are largely attributable to the generalized adrenergic activation, affecting neuronal activity and neurotransmitter balance. The presence of these neurological symptoms, especially when disproportionate to the primary respiratory condition, should prompt consideration of salbutamol toxicity in the differential diagnosis.

The coexistence of hypothyroidism in an asthmatic patient receiving salbutamol introduces a complex interplay of physiological factors that can modulate the drug's effects and toxicological profile. Thyroid hormones exert a profound influence on adrenergic receptor density and sensitivity throughout the body [9]. Specifically, an inverse relationship exists between the level of thyroid function and airway β-adrenergic responsiveness [9]. In hypothyroid individuals, a reduction in airway β-adrenergic responsiveness has been observed, manifesting as a significant decrease in specific airway conductance after salbutamol administration, along with a reduction in the area under the salbutamol dose-response curve [9].

This diminished responsiveness implies that a hypothyroid patient may require higher doses of salbutamol to achieve adequate bronchodilation, potentially leading to a therapeutic challenge. If clinicians attempt to overcome this reduced efficacy by escalating salbutamol doses, the risk of systemic toxicity increases, even if the primary bronchial β2-receptors are less responsive. The systemic β2-receptors, particularly those influencing cardiac and metabolic parameters, might still respond to elevated circulating salbutamol concentrations, or their sensitivity may be affected differently by thyroid status. Consequently, a hypothyroid asthmatic female, who may already experience challenges with asthma control, could be particularly susceptible to the systemic adverse effects of salbutamol if dosing is not carefully adjusted to her altered adrenergic sensitivity. This necessitates a heightened awareness of potential toxicity even at doses that might be considered standard for euthyroid individuals.

Diagnosing salbutamol toxicity in a young female with coexisting asthma and hypothyroidism requires a comprehensive approach, integrating clinical presentation with laboratory findings. The clinical picture often involves a constellation of symptoms disproportionate to the underlying asthma exacerbation. These include persistent tachycardia, palpitations, tremor, restlessness, and possibly paradoxical hypotension [8]. A thorough medication history, specifically detailing the total dose, frequency, and route of salbutamol administration, is paramount.

Laboratory investigations are crucial for confirming and quantifying the toxicity. Key markers to assess include serum potassium, which is typically depressed due to intracellular shifts [8]. Blood glucose levels should be monitored for hyperglycemia [8]. Additionally, assessment of lactate levels can reveal lactic acidosis. An ECG is essential to detect cardiac rhythm disturbances, such as prolonged QTc interval or T-wave changes. In the context of hypothyroidism, thyroid function tests (thyroid-stimulating hormone and free T4) should be reviewed to understand the baseline endocrine status and its potential contribution to altered adrenergic responsiveness. Differentiating between severe asthma symptoms and salbutamol toxicity can be challenging, as both can present with dyspnea and tachycardia. However, the presence of significant electrolyte imbalances or profound metabolic derangements strongly points towards toxicity. Plasma salbutamol levels, if available, can support the diagnosis but are often not immediately accessible in acute settings.

Management of salbutamol toxicity prioritizes discontinuation of the offending agent and supportive care to mitigate systemic effects. Upon recognizing toxicity, immediate cessation of salbutamol administration is the primary intervention. This allows for the natural metabolism and excretion of the drug, leading to resolution of symptoms over time, typically within 24 hours.

Supportive measures specifically address the metabolic and cardiovascular derangements. Hypokalemia, a common and potentially life-threatening complication, requires prompt IV potassium supplementation, guided by frequent electrolyte monitoring. Hyperglycemia usually resolves with cessation of salbutamol, but insulin administration may be necessary for severely elevated glucose levels. Fluid management is important to maintain hydration and support renal elimination. For significant tachycardia or cardiac arrhythmias, careful consideration of β-adrenergic receptor antagonists (beta-blockers) may be necessary. However, their use in asthmatic patients is complex due to the risk of exacerbating bronchospasm by blocking beneficial β2-receptors in the airways. Cardioselective β1-blockers might be considered, but only with extreme caution and continuous cardiorespiratory monitoring. In cases of severe lactic acidosis, bicarbonate administration might be employed, though addressing the underlying cause (salbutamol cessation) is paramount. Continuous monitoring of vital signs, ECG, and serial laboratory parameters (electrolytes, glucose, and lactate) guides the therapeutic approach and assesses response to interventions. Given the patient's hypothyroid status, careful attention to fluid and electrolyte balance is particularly important, as thyroid dysfunction can independently influence these parameters.

Salbutamol remains a vital therapeutic agent for asthma management, yet its systemic pharmacodynamics necessitate careful dosing to prevent toxicity. For a young asthmatic female also affected by hypothyroidism, the risk-benefit profile of salbutamol becomes nuanced. The inverse relationship between thyroid function and β-adrenergic responsiveness means that hypothyroid individuals may exhibit a reduced bronchodilatory response to standard doses, potentially leading to dose escalation and an elevated risk of systemic adverse effects.

Salbutamol toxicity manifests through a range of cardiovascular, metabolic, and neurological complications, including tachycardia, hypokalemia, hyperglycemia, lactic acidosis, and tremor. Accurate diagnosis relies on a combination of clinical suspicion, detailed medication history, and crucial laboratory assessments. Management centers on prompt discontinuation of the drug and meticulous supportive care, focusing on correcting electrolyte imbalances, monitoring cardiac function, and addressing metabolic disturbances. The unique physiological context of coexisting hypothyroidism demands heightened clinical vigilance, reinforcing the need for individualized therapeutic strategies and close monitoring to ensure both effective asthma control and patient safety.

## Conclusions

Salbutamol is an uncommon drug of abuse, though it is often misused by patients with poorly controlled asthma for its bronchodilatory effects. The diagnosis of salbutamol toxicity relies on a thorough clinical history and symptom assessment, supported by laboratory findings such as hyperglycemia, hypokalemia, and metabolic or lactic acidosis. Our patient exhibited the classic features of inadvertent salbutamol toxicity. This case highlights the importance of patient education regarding the correct use of salbutamol to prevent recurrence of such events. The patient was managed conservatively and advised to maintain regular follow-up with her treating physicians.
